# Focal Correction of Severe Fixed Kyphosis with Single Level Posterior Ponte Osteotomy and Interbody Fusion

**DOI:** 10.7759/cureus.653

**Published:** 2016-06-23

**Authors:** Seth S Molloy, Faiz U Ahmad, Griffin R Baum, Barth A Green, Nathan H Lebwohl

**Affiliations:** 1 Neurosurgery, Palmetto Health Associates; 2 Department of Neurological Surgery, Emory University School of Medicine; 3 Neurosurgery, University of Miami

**Keywords:** kyphosis, spine, spine deformity, scoliosis correction, ponte, interbody fusion, infections, scheuermann’s kyphosis

## Abstract

Objective: To report the successful correction of a severe, fixed kyphotic deformity utilizing a combination posterior lumbar interbody fusion (PLIF) and Ponte osteotomy at the site of acute kyphosis.

Summary of Background Data: There have been no reports on the experience and surgical strategy of combined one-level focal PLIF and Ponte osteotomy for fixed severe kyphotic deformity. Typically, these corrections would need a pedicle subtraction osteotomy or a vertebrectomy.

Methods: A 24-year-old man presented with progressive back pain and a fixed severe thoracolumbar kyphosis centered at the L2-L3 disc space seven years after removal of instrumentation for intractable infection following correction of Scheuermann's Kyphosis. The patient also demonstrated pseudoarthrosis of the posterior thoracolumbar fusion bed. The original operative plan was to perform a vertebral column resection (VCR) of L2 to correct his severe kyphosis.  During preparation for the VCR, the patient’s deformity corrected completely after insertion of blunt distraction paddles for the interbody fusion after the Ponte osteotomy at L2-L3. A VCR was avoided, and the construct was able to be completed with simple rod insertion and posterolateral fusion.

Results: The described technique achieved 69 degrees of correction at the L2-L3 disc space without any remodeling of the surrounding vertebrae. The C7 plumb line was normalized, and the patient was able to stand upright with horizontal gaze and without pre-existing discomfort. At the six-month follow-up, the patient reported a significant improvement in pain and was able to resume normal activities.

## Introduction

Severe focal thoracolumbar kyphotic deformities often require a high grade osteotomy either in the form of a pedicle subtraction osteotomy (PSO) or a vertebrectomy (VCR) for effective correction of the deformity. We describe a case of a young man with Scheuermann’s Kyphosis who underwent a 69 degree correction using a combination of Ponte osteotomy and posterior lumbar interbody fusion (PLIF) without the use of a high grade osteotomy technique. The patient agreed to participate and was explained the nature and objectives of this study, and informed consent was formally obtained. No reference to the patient's identity was made at any stage during data analysis or in the report. 

## Case presentation

A 24-year-old white man presented to the authors institution with progressive kyphosis and severe back pain.  The patient had undergone a previous thoracic fusion operation at the age of 17 for Scheuermann's Kyphosis, which was complicated by a postoperative infection requiring hardware removal and brace immobilization for approximately 1.5 years. Exertion, lifting, and attempting to maintain horizontal gaze worsened his symptoms while rest was the only method to improve his pain.

**Figure 1:**
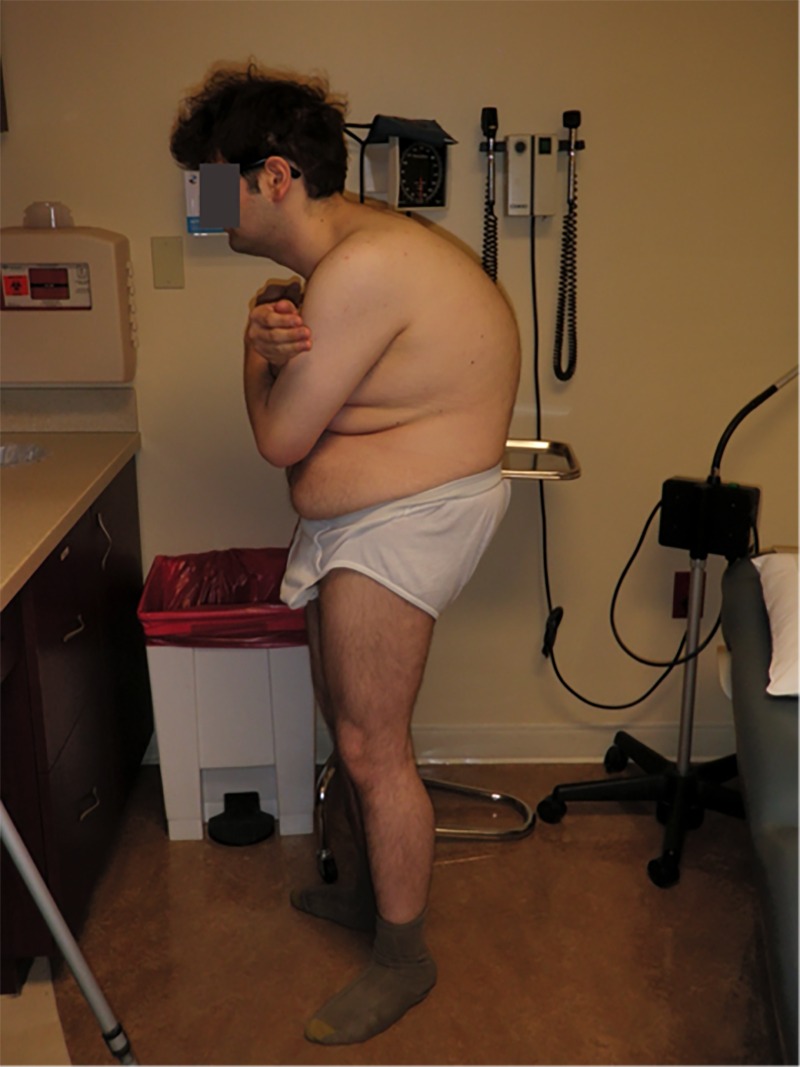
Preoperative Clinical Photograph Lateral photograph demonstrating standing posture and kyphosis impairing horizontal gaze

On physical examination, the patient was 5 feet 5 inches, 190 lbs., and demonstrated severe thoracolumbar kyphosis (Figure 1). He had well healed operative scars of the posterior midline as well as a right thorax. His neurological exam was normal. Deep tendon reflexes and straight leg raising tests were all normal.

**Figure 2:**
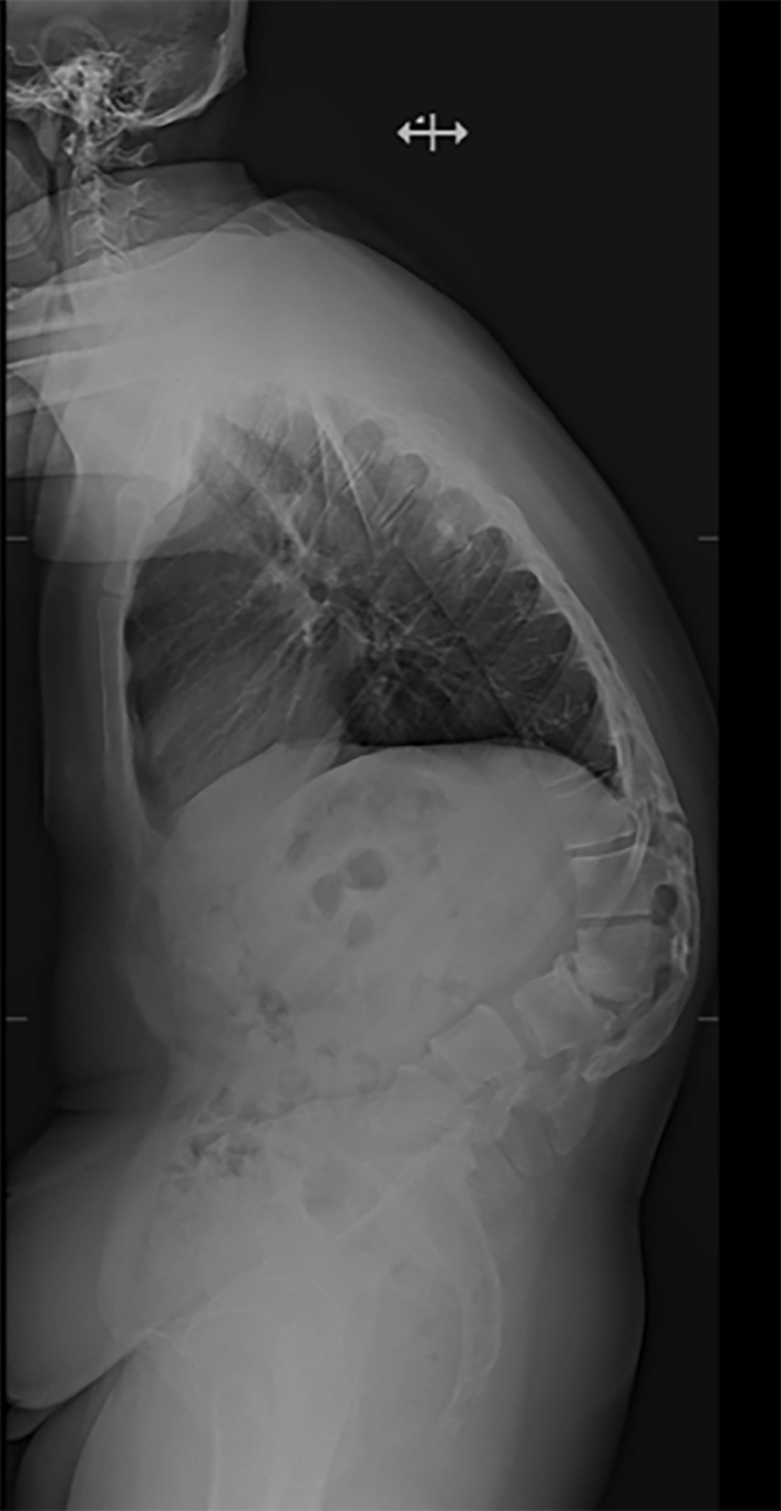
Preoperative Lateral Radiograph Preoperative lateral radiograph demonstrating standing posture and acute kyphosis at L2-L3

**Figure 3:**
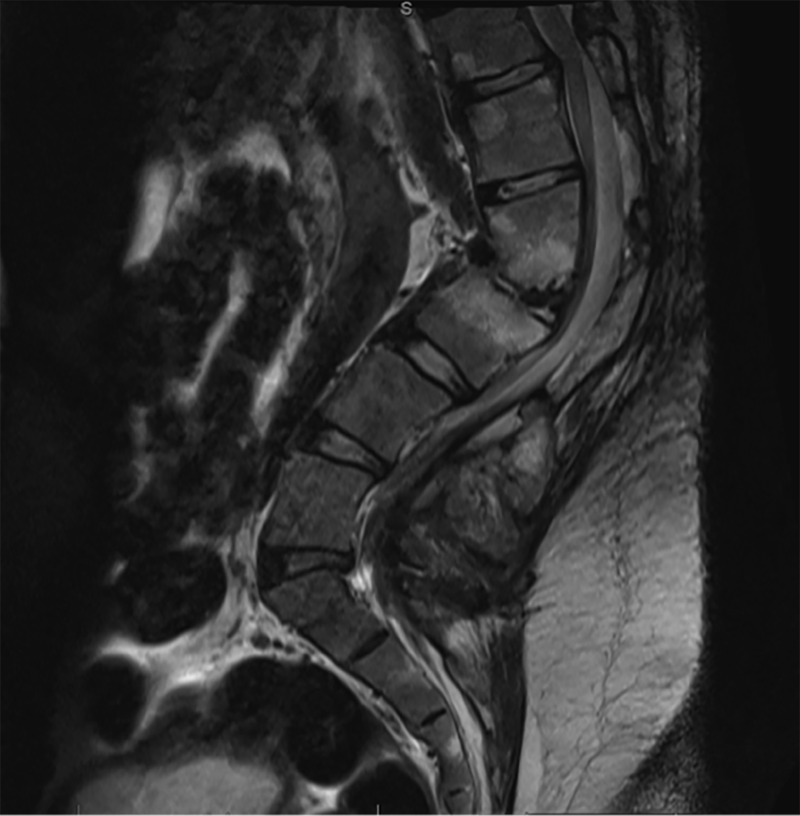
Preoperative Sagittal MRI T2 weighted sagittal MR image demonstrating kyphosis at L2-L3 and spinal stenosis at the area of the pseudoarthrosis

Imaging revealed severe focal lumbar kyphosis of 69 degrees at L2-L3 (Figure 2). There was evidence of a robust posterior fusion mass from T10-T12 and from L1-S1 with pseudoarthrosis and spinal stenosis at the T12-L1 level (Figure 3).

The original operative plan was T4-pelvis instrumentation with a Ponte osteotomy at L2 and laminectomy at T12-L1 for stenosis at the area of pseudoarthrosis. This was to be followed by a posterior vertebral column resection (VCR) of L2 for correction of the deformity. The operation commenced with the placement of pedicle screw instrumentation from T5 to S1, pelvic fixation using standard iliac screw technique, and finally with transverse process hooks at T4. Pedicle screws were placed above and below the level of pseudoarthrosis at T12-L1 and originally left out of L2 in preparation for the VCR. A functional laminectomy with removal of fibrocartilaginous scar at T12-L1 was then performed. A Ponte osteotomy was completed at the L2-L3 disc space, and preparation for an interbody fusion started.

**Figure 4:**
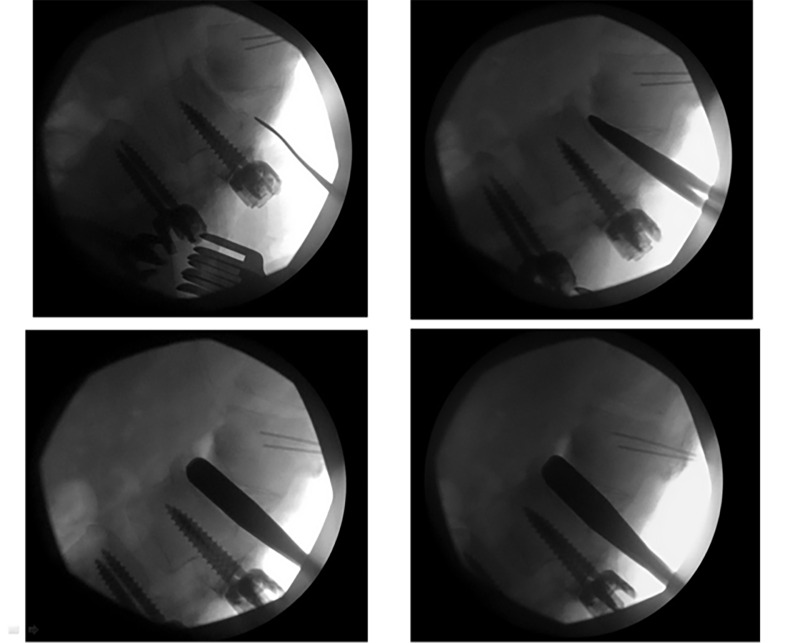
Intraoperative Fluoroscopic Images Intraoperative fluoroscopic images demonstrating sequential dilation technique and correction of kyphosis using Ponte osteotomy and interbody fusion technique

Once the blunt paddles were placed within the disc space and sequentially dilated, the space became mobile, and a complete correction of deformity was demonstrated on intraoperative fluoroscopy (Figure 4).

**Figure 5:**
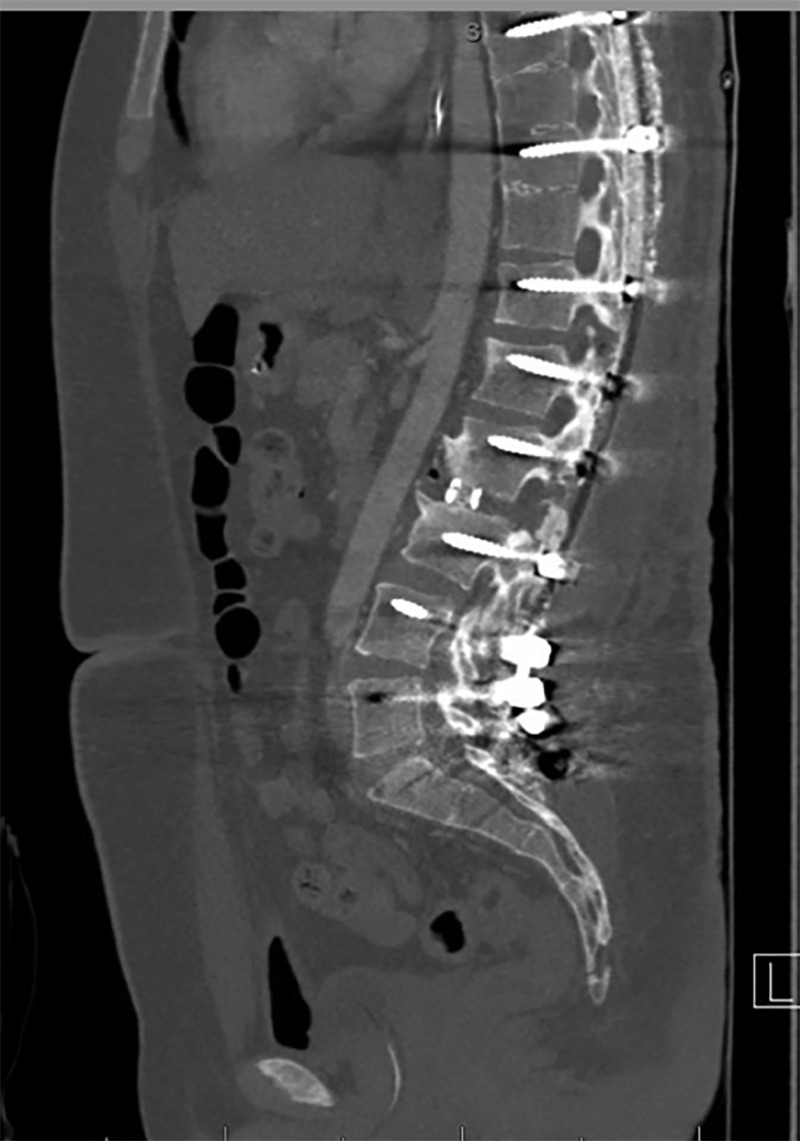
Postoperative Sagittal CT Image Immediate postoperative sagittal CT image demonstrating satisfactory placement of hardware, and complete correction of kyphotic deformity using Ponte osteotomy and posterior interbody fusion technique

Two titanium cages were filled with autograft bone and implanted in the disc space. Additional screws were then implanted into L2, and two titanium rods were contoured to provide maximum lordosis over the corrected spine followed by placement of the bone graft (Figure 5) in the interlaminar spaces as well as the intertransverse gutters. Due to the intact laminae at L5 and S1 enabling a large surface area for interlaminar fusion of the L5-S1 segment, it was not felt that an L5-S1 interbody graft was necessary (as has been recommended in long segment thoracolumbar fusions).

The patient experienced a postoperative ileus that resolved in four days with conservative measures. A CT angiogram of the abdomen was performed and did not support any acute abdominal pathology or mesenteric ischemia. The patient did not experience any other perioperative complications.

**Figure 6:**
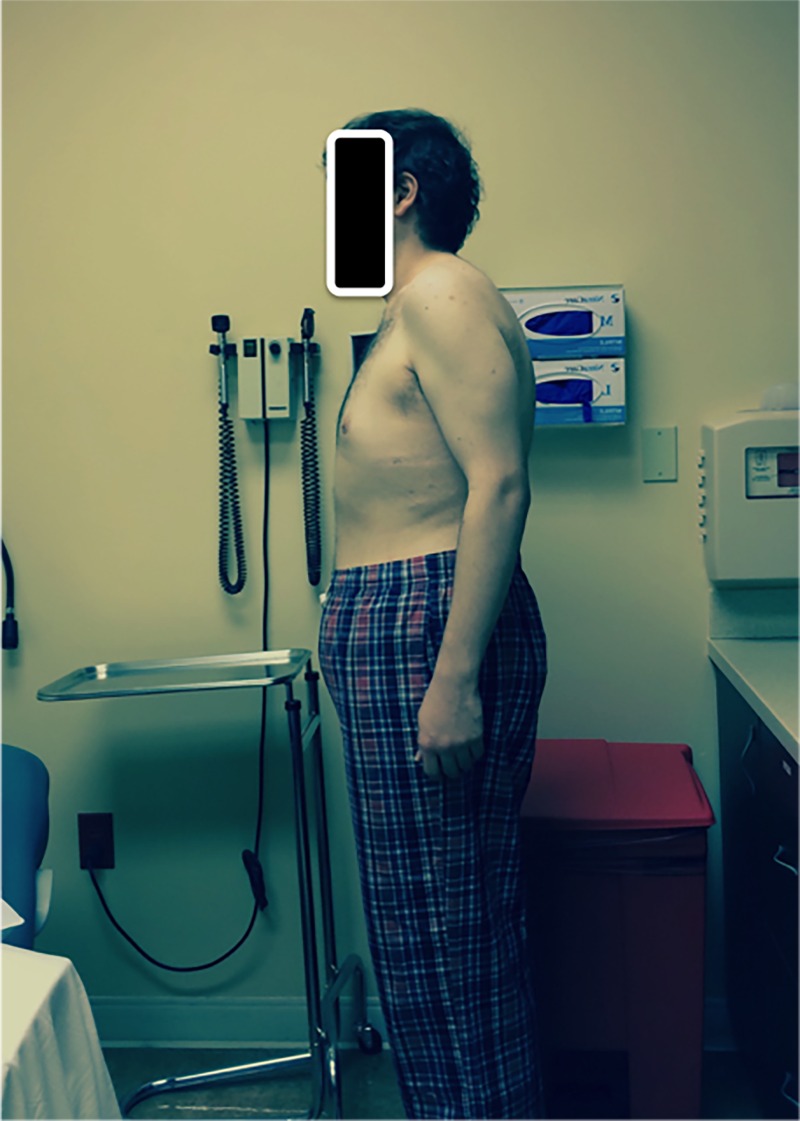
Postoperative Clinical Photograph Postoperative Lateral Clinical Photograph demonstrating correction of both kyphosis and impaired horizontal gaze

The patient was discharged on postoperative day eight. At the six-month follow-up, his posture was normalized, and he regained horizontal gaze, helping him achieve ambulation without the use of his previous walking device (Figure 6).

## Discussion

Thoracolumbar kyphotic deformities can be corrected by Smith-Peterson or multi-segmental chevron osteotomies, with most severe sagittal deformities benefiting from additional vertebral reconstruction in the form of either a pedicle subtraction osteotomy (PSO) or VCR. [1-2]. Schwab et al., described a comprehensive anatomical spinal osteotomy classification system correlating stepwise bony removal to stepwise grades of osteotomies [3]. We initially planned to do a VCR or a Grade 5 anatomic resection at L2 to optimize correction from a single level, considering the health of the remaining spine and posterior fusion bed. However, it was recognized that when the collapsed L2-L3 disc space was reconstituted using sequential bilateral dilation, the patient slowly corrected in the sagittal plane to normal alignment, negating the need for further intervention beyond a Grade 2 anatomic resection of the posterior vertebral column. Since the patient had a concurrent pseudoarthrosis at T12-L1, the instrumentation was inserted from T4-pelvis to maximize fixation points and stability over a rather large, healed fusion bed.

Even though this case is unique in its presentation, the concept of correcting this type of deformity through a combination of simple osteotomies and interbody mobilization could have additional applications, particularly in those patients with sufficient bone quality and no clear contraindications to a gross correction. Kanayama recently described a similar technique in a series of older patients where he used interbody spacers on each side of a PSO to help create an effective bone fusion environment post-correction [4]. Even though proven quite effective, the PSO increases the risk of morbidity including excessive blood loss, higher complication rate, increased risk of pseudoarthrosis, and a steep learning curve for the surgeon. The described approach may have a slight advantage in that it preserves the height of the vertebral column without morbidity of an additional corpectomy. The cage also provides an osseous conduit for a solid bony fusion to form in the area of focal degeneration, helping alleviate the future potential for failure. 

Another consideration that the authors were reminded of with this case was the global shift of intra-abdominal contents and the associated complications following correction of an acute kyphosis. Abdominal compartment syndrome, mesenteric ischemia, and duodenal obstruction are some of the most severe abdominal complications and should be quickly recognized in the postoperative setting, with close clinical examination and radiographic imaging [5]. Fortunately, our patient had complete resolution of his symptoms without any additional intervention, but this may not always be the case, and every surgeon targeting such a significant correction goal should keep these complications in mind during the postoperative period.

## Conclusions

In conclusion, the authors present a case of a severe, fixed focal kyphotic deformity corrected through a single disc space approach via a Ponte osteotomy and interbody fusion. This technique spared the patient the additional morbidity of a full vertebral body resection and added operative time, while potentially providing a more balanced fixation construct most reflective of his pre-kyphotic state. To the authors' knowledge, this is the first report to describe such magnitude of correction (>60 degrees) through a single disc space. 
